# Analysis of North Carolina Medicaid Claims Data to Simulate a Pediatric Accountable Care Organization

**DOI:** 10.1001/jamanetworkopen.2023.27264

**Published:** 2023-08-04

**Authors:** Rushina Cholera, David M. Anderson, Richard Chung, Jessica Genova, Peter Shrader, William K. Bleser, Robert S. Saunders, Charlene A. Wong

**Affiliations:** 1 Duke Margolis Center for Health Policy, Duke University, Durham, North Carolina; 2 Duke Department of Pediatrics, Duke University, Durham, North Carolina; 3 Duke Department of Population Health Sciences, Duke University, Durham, North Carolina

## Abstract

**Importance:**

Despite momentum for pediatric value-based payment models, little is known about tailoring design elements to account for the unique needs and utilization patterns of children and young adults.

**Objective:**

To simulate attribution to a hypothetical pediatric accountable care organization (ACO) and describe baseline demographic characteristics, expenditures, and utilization patterns over the subsequent year.

**Design, Setting, and Participants:**

This retrospective cohort study used Medicaid claims data for children and young adults aged 1 to 20 years enrolled in North Carolina Medicaid at any time during 2017. Children and young adults receiving at least 50% of their primary care at a large academic medical center (AMC) in 2017 were attributed to the ACO. Data were analyzed from April 2020 to March 2021.

**Main Outcomes and Measures:**

Primary outcomes were total cost of care and care utilization during the 2018 performance year.

**Results:**

Among 930 266 children and young adults (377 233 children [40.6%] aged 6-12 years; 470 612 [50.6%] female) enrolled in Medicare in North Carolina in 2017, 27 290 children and young adults were attributed to the ACO. A total of 12 306 Black non-Hispanic children and young adults (45.1%), 6308 Hispanic or Latinx children and young adults (23.1%), and 6531 White non-Hispanic children and young adults (23.9%) were included. Most attributed individuals (23 133 individuals [84.7%]) had at least 1 claim in the performance year. The median (IQR) total cost of care in 2018 was $347 ($107-$1123); 272 individuals (1.0%) accounted for nearly half of total costs. Compared with children and young adults in the lowest-cost quartile, those in the highest-cost quartile were more likely to have complex medical conditions (399 individuals [6.9%] vs 3442 individuals [59.5%]) and to live farther from the AMC (median [IQR distance, 6.0 [4.6-20.3] miles vs 13.9 [4.6-30.9] miles). Total cost of care was accrued in home (43%), outpatient specialty (19%), inpatient (14%) and primary (8%) care. More than half of attributed children and young adults received care outside of the ACO; the median (IQR) cost for leaked care was $349 ($130-$1326). The costliest leaked encounters included inpatient, ancillary, and home health care, while the most frequently leaked encounters included behavioral health, emergency, and primary care.

**Conclusions and Relevance:**

This cohort study found that while most children attributed to the hypothetical Medicaid pediatric ACO lived locally with few health care encounters, a small group of children with medical complexity traveled long distances for care and used frequent and costly home-based and outpatient specialty care. Leaked care was substantial for all attributed children, with the cost of leaked care being higher than the total cost of care. These pediatric-specific clinical and utilization profiles have implications for future pediatric ACO design choices related to attribution, accounting for children with high costs, and strategies to address leaked care.

## Key Points

**Question:**

What are the baseline demographic characteristics, cost, and utilization patterns among children and young adults attributed to a hypothetical pediatric Medicaid accountable care organization (ACO)?

**Findings:**

In this cohort study including 27 290 children and young adults prospectively attributed to a hypothetical ACO, a small group of individuals with medical complexity accounted for more than half of the total cost of care, with most cost accruing in home-based and outpatient specialty encounters. More than half of children and young adults sought care outside of the ACO.

**Meaning:**

This cohort study found costly home-based and outpatient specialty care utilization for a small population of children with medical complexity and frequent out-of-ACO care for all attributed children.

## Teaser

This cohort study simulates attribution of North Carolina Medicaid recipients aged 1 to 20 years to a pediatric accountable care organization to describe baseline demographic characteristics, expenditures, and utilization patterns over the subsequent year.

## Introduction

Value-based alternative payment models (APMs) intend to reward care quality and efficiency rather than volume of services and have expanded rapidly.^1,2,3,4,5^ Accountable care organizations (ACOs) are the most widespread APM, in which health care groups assume responsibility for care quality and costs for a defined patient population, with potential to share savings and sometimes losses. The US government implemented multiple Medicare ACOs through the Patient Protection and Affordable Care Act, with modest successes.^5^ There is increasing investment in ACOs that account for the unique needs of children.^6^ For example, the Center for Medicare and Medicaid Innovation Integrated Care for Kids model has funded 7 nationwide pilot programs to test the outcomes associated with integrated health care delivery models supported by child-focused Medicaid APMs.^7^

Despite increasing interest, there is limited evidence on strategies that contribute to successful pediatric ACOs. Given fundamental differences in child vs adult populations, the design of child-focused ACOs requires thoughtful recalibration of adult-oriented models.^8,9,10^ Many adult-focused ACOs focus on short-term cost savings by reducing postacute care or hospitalization for chronic disease. However, most children are healthy and a small proportion ever require hospitalization, necessitating different cost saving targets. Child-focused models can benefit from a life-course framework emphasizing prevention and healthy development, with metrics accounting for long-term impacts on health.^11^ Pediatric models might also consider incorporating familial and education contexts, given their profound influence during childhood.^10,11,12,13^

Early pediatric ACOs have shown promise in increasing preventive services and reducing costs.^14,15,16,17,18^ However, designs have varied widely, with few published reports. Thus, it is difficult to identify components that influence pediatric ACO success.^19,20^ To inform development of child-focused ACOs, we used Medicaid claims data from an academic medical center (AMC) to simulate attribution to an ACO for Medicaid-enrolled children and young adults. We then described utilization patterns and expenditures over the subsequent performance year. By assessing the baseline care patterns of children and young adults attributed to the hypothetical ACO, we sought to preemptively identify targets that might be sensitive to policy levers and population health management strategies within pediatric ACOs.

## Methods

This cohort study was determined to be exempt from review and informed consent by the Duke University institutional review board. This study adheres to the Strengthening the Reporting of Observational Studies in Epidemiology (STROBE) reporting guideline for observational studies.

### Data and Study Population

Medicaid-insured children and young adults in North Carolina who received primary care at 1 AMC were attributed to the simulated ACO. The AMC offers inpatient and most pediatric subspecialty services and has an extensive network of outpatient facilities statewide. State Medicaid physical and behavioral health claims from January 1, 2017, to December 31, 2018, were included in analysis. Children and young adults aged 1 to 20 years as of December 31, 2017, and enrolled in Medicaid at any time during 2017 were included. Children younger than age 1 year were excluded due to unique attribution complexities and utilization trajectories of the newborn period.^21^ Claims data from 2017 were used to attribute beneficiaries to the ACO. Claims for services in 2018 data were used to analyze ACO performance with respect to cost and utilization patterns.

### Patient Characteristics

Sociodemographic variables from Medicaid enrollment data included patient age, race, ethnicity, and last county of residence in 2017. Race and ethnicity were extracted from Medicaid claims in which beneficiaries self-report race and ethnicity, categorized as Black non-Hispanic, Hispanic or Latinx, multiple races or other non-Hispanic ethnicity (including American Indian, Asian, Hawaiian or Pacific Islander, or multiple races), White non-Hispanic, and unknown. We included both race and ethnicity in analysis to allow for a better understanding of how multilevel structural discrimination can lead to differential outcomes for minoritized populations. Urban or rural status was defined using Rural-Urban Commuting Area Codes based on the enrollee’s home zip code.^22^ Distance from AMC campus was calculated using the centroids of enrollees’ home zip codes and the central AMC campus. Medical complexity was categorized using the Pediatric Medical Complexity Algorithm (PMCA) using all claims from 2016 to 2018. Children and young adults were categorized into mutually exclusive groups: (1) healthy, without chronic disease, (2) noncomplex chronic disease, and (3) complex chronic disease.^23^

### Attribution

**Figure 1.**
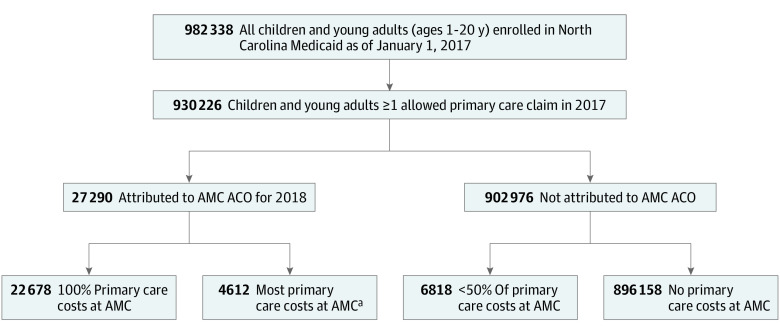
Simulation of Prospective Attribution to the Hypothetical Medicaid Accountable Care Organization (ACO) Based on Prior Year Primary Care Usage AMC indicates academic medical center. ^a^Defined as at least 50% but less than 100%.

At the time of analysis, North Carolina was transitioning to Medicaid managed care. The state required value-based payment arrangements in contracting with managed care organizations, with payments structured to flow through managed care organizations to ACOs. Attribution decisions were based on North Carolina’s planned ACO design and review of methods used by existing ACOs.^24,25^ To align with North Carolina’s plan to base attribution on primary care assignment, children and young adults were prospectively attributed using primary care costs from 2017 (Figure 1). The AMC was identified by matching Tax Identification Numbers and associated Type 2 Facility National Provider Identifiers (NPIs). Health care specialty was retrieved from Type 1 NPI taxonomy codes. Children and young adults with any nonzero-dollar primary care claim performed by a clinician with a primary care practitioner (PCP) NPI taxonomy at the AMC in 2017 were identified using Healthcare Common Procedure Coding System and *Current Procedure Terminology* version 4 codes not at an urgent care or retail clinic. Children with at least 50% of primary care costs at the AMC were attributed.

### Statistical Analysis

We calculated total expenditures in 2018 for attributed children. Similar to Medicare Shared Savings Program (MSSP) methods,^26^ total cost of care (TCOC) was defined as the sum of total allowed amount for all health care encounter claims during the performance year, excluding pharmacy claims. Costs were not inflation adjusted. For children and young adults with at least 1 claim, TCOC was categorized by quartile (Q), with Q1 indicating the lowest cost and Q4, highest. Within each quartile, sociodemographic characteristics and Medicaid enrollment duration were examined. We also assessed cost of services, including primary care, imaging, ancillary services (eg, physical, speech, occupational therapies), and behavioral health.

We examined care utilization patterns across inpatient, emergency department, urgent care, dental care, outpatient primary care, outpatient specialty care, and home care settings. Settings and service types were defined by a combination of Healthcare Common Procedure Coding System codes, revenue, place of service, and NPI taxonomy codes. We quantified care received outside of the ACO as leaked care.

Sensitivity analyses were conducted to compare attribution methods, prescription drug cost inclusion, and outlier exclusion. *P* values were 2-sided, and statistical significance was set at *P* = .05. Analyses were performed using SAS version 9.4 (SAS Institute). Data were analyzed between April 2020 and March 2021.

## Results

### Attribution Beneficiary Characteristics

In 2017, 930 266 children and young adults (377 233 children [40.6%] aged 6-12 years; 470 612 [50.6%] female) were primarily insured by North Carolina Medicaid and had at least 1 claim. Individuals with all (22 678 individuals) or most (defined as ≥50% but <100%; 4612 individuals) of their primary care costs at the AMC were attributed (Table 1). Characteristics of attributed children and young adults included mean (SD) age 10.3 (5.4) years, with 13 694 (50.2%) female; 12 306 children and young adults (45.1%) were Black non-Hispanic, 6308 children and young adults (23.1%) were Hispanic or Latinx, and 6531 children and young adults (23.9%) were White non-Hispanic. Most individuals were income-eligible for Medicaid (23 999 individuals [87.9%]) while 2654 individuals (9.7%) were in the blind or disabled category, and 589 individuals (2.2%) were eligible due to foster care engagement. Annual primary care spending in 2017 was lower for children and young adults attributed by 100% PCP costs (median [IQR], $160 [$98-$280]) compared with those attributed by most PCP costs (median [IQR], $307 [$169-$554]). Individuals with 100% PCP costs within the ACO, compared with those attributed by most PCP costs, were less likely to remain continually enrolled in Medicaid (20 261 individuals [89.3%] vs 4309 individuals [93.4%]), lived closer to the AMC (median [IQR] distance, 6.0 [4.6-20.3] miles vs 26.1 [6.0-45.0] miles), and were less likely to have medically complex conditions (4264 individuals [18.8%] vs 2209 individuals [47.9%]).

**Table 1.** Characteristics of Children and Young Adults Enrolled in North Carolina Medicaid by Accountable Care Organization Attribution Status

Characteristic	Individuals, No. (%)				
	All 2017 enrollees with ≥1 claim (N = 930 266)	Attributed		Not attributed	
		All cost at AMC (n = 22 678)^a^	>50% Cost at AMC (n = 4612)	<50% Cost at AMC (n = 6818)	No costs at AMC (n = 896 158)
2017 Primary care cost at AMC					
Median (IQR), %	0 (0-0)	100 (100-100)	72 (60-84)	25 (14-37)	0 (0-0)
2017 Primary care cost at AMC, Mean (SD), %	3.0 (16.4)	100 (0)	72.5 (14.0)	25.6 (13.1)	0 (0)
Median (IQR), $	0 (0-0)	160 (98-280)	307 (169-554)	96 (62-160)	0 (0-0)
Mean (SD), $	9.6 (125.26)	246.3 (432.54)	518.3 (1281.45)	135.7 (135.87)	0 (0)
Continually enrolled	836 975 (90.0)	20 261 (89.3)	4309 (93.4)	6399 (93.9)	806 006 (89.9)
Medicaid eligibility					
General pediatrics	861 310 (92.6)	20 503 (90.4)	3496 (75.8)	5290 (77.6)	832 021 (92.8)
Foster care	19 520 (2.1)	465 (2.1)	124 (2.7)	253 (3.7)	18 678 (2.1)
Blind or disabled	47 034 (5.1)	1672 (7.4)	982 (21.3)	1264 (18.5)	43 116 (4.8)
Sex					
Male	459 654 (49.4)	11 276 (49.7)	2320 (50.3)	3537 (51.9)	442 521 (49.4)
Female	470 612 (50.6)	11 402 (50.3)	2292 (49.7)	3281 (48.1)	453 637 (50.6)
Age group, y					
1-5	247 074 (26.6)	6334 (27.9)	1298 (28.1)	1825 (26.8)	237 617 (26.5)
6-12	377 233 (40.6)	9168 (40.4)	1586 (34.4)	2574 (37.8)	363 905 (40.6)
13-17	216 439 (23.3)	4840 (21.3)	1137 (24.7)	1703 (25.0)	208 759 (23.3)
18-20	89 520 (9.6)	2336 (10.3)	591 (12.8)	716 (10.5)	85 877 (9.6)
Race and ethnicity					
Black non-Hispanic	294 135 (31.6)	10 715 (47.2)	1591 (34.5)	2180 (32.0)	279 649 (31.2)
Hispanic or Latinx	201 961 (21.7)	5391 (23.8)	917 (19.9)	1422 (20.9)	194 231 (21.7)
Multiple or other non-Hispanic^b^	61 879 (6.7)	1169 (5.2)	278 (6.0)	416 (6.1)	60 016 (6.7)
White non-Hispanic	353 072 (38.0)	4901 (21.6)	1630 (35.3)	2569 (37.7)	343 972 (38.4)
Unknown	19 219 (2.1)	502 (2.2)	196 (4.2)	231 (3.4)	18 290 (2.0)
Rural residence	232 713 (25.0)	2489 (11.0)	906 (19.6)	1168 (17.1)	228 150 (25.5)
Distance from AMC, median (IQR), mi	90.0 (55.6-124.4)	6.0 (4.6-20.3)	26.1 (6.0-45.0)	29.2 (21.1-57.3)	92.1 (59.4-124.6)
PMCA					
Nonchronic	526 525 (56.6)	12 173 (53.7)	1352 (29.3)	1855 (27.2)	511 145 (57.0)
Noncomplex chronic	256 192 (27.5)	6241 (27.5)	1051 (22.8)	1826 (26.8)	247 074 (27.6)
Complex chronic	147 549 (15.9)	4264 (18.8)	2209 (47.9)	3137 (46.0)	137 939 (15.4)

Abbreviations: AMC, academic medical center; PMCA, Pediatric Medical Complexity Algorithm.

^a^ Cost refers to primary care cost.

^b^ Includes American Indian, Asian, and Hawaiian or Pacific Islander.

### ACO Performance

#### Medicaid Coverage

Most individuals (23 582 individuals [86.4%]) remained continually enrolled in the performance year (Table 2). The 13.6% of children and young adults enrolled for less than 12 months were more likely to be ages 18 to 20 years and White non-Hispanic compared with continually enrolled children. A total of 987 attributed individuals (3.6%) and young adults had no Medicaid enrollment in 2018.

**Table 2.** Characteristics by Cost Quartile in 2018 Among All Attributed Children and Young Adults

Characteristic	Individuals, No. (%)				
	Attributed population (N = 27 290)^a^	Cost quartile 1 (n = 5782)	Cost quartile 2 (n = 5784)	Cost quartile 3 (n = 5784)	Cost quartile 4 (n = 5783)
Total cost, median (IQR), $	347 (107-1123)	110 (82-155)	306 (247-381)	771 (599-1025)	3844 (2224-8006)
Primary care cost at AMC in 2017, %					
100	22 678 (83.1)	5262 (91.0)	5105 (88.3)	4687 (81.0)	3866 (66.9)
≥50	4612 (16.9)	520 (9.0)	679 (11.7)	1097 (19.0)	1917 (33.1)
Continually enrolled	23 582 (86.4)	5076 (87.8)	5311 (91.8)	5451 (94.2)	5512 (95.3)
Medicaid eligibility					
Income-eligible (general pediatric)	23999 (87.9)	5498 (95.1)	5405 (93.4)	5216 (90.2)	3934 (68.0)
Foster care	589 (2.2)	99 (1.7)	120 (2.1)	125 (2.2)	182 (3.1)
Blind or disabled	2654 (9.7)	181 (3.1)	255 (4.4)	438 (7.6)	1642 (28.4)
Sex					
Male	13 596 (49.8)	2909 (50.3)	2782 (48.1)	2738 (47.3)	2970 (51.4)
Female	13 694 (50.2)	2873 (49.7)	3002 (51.9)	3046 (52.7)	2813 (48.6)
Age category, y					
1-5	7632 (28.0)	1704 (29.5)	1706 (29.5)	1618 (28.0)	1738 (30.1)
6-12	10 754 (39.4)	2562 (44.3)	2499 (43.2)	2277 (39.4)	1891 (32.7)
13-17	5977 (21.9)	1144 (19.8)	1206 (20.9)	1406 (24.3)	1352 (23.4)
18-21	2927 (10.7)	372 (6.4)	373 (6.4)	483 (8.4)	802 (13.9)
Race and ethnicity					
Black non-Hispanic	12 306 (45.1)	2623 (45.4)	2631 (45.5)	2585 (44.7)	2480 (42.9)
Hispanic or Latinx	6308 (23.1)	1578 (27.3)	1468 (25.4)	1384 (23.9)	1053 (18.2)
Multiple/other non-Hispanic^b^	1447 (5.3)	305 (5.3)	318 (5.5)	280 (4.8)	340 (5.9)
White non-Hispanic	6531 (23.9)	1188 (20.5)	1273 (22.0)	1416 (24.5)	1627 (28.1)
Unknown	698 (2.6)	88 (1.5)	94 (1.6)	119 (2.1)	283 (4.9)
Rural	3395 (12.4)	582 (10.1)	677 (11.7)	827 (14.3)	836 (14.5)
Distance from AMC, median (IQR), mi	6.0 (4.6, 26.0)	6.0 (4.6, 20.3)	6.0 (4.6, 23.9)	6.0 (4.6, 26.0)	13.9 (4.6, 30.9)
PMCA					
Nonchronic	13 262 (48.3)	4113 (71.1)	3378 (58.4)	2290 (39.6)	850 (14.7)
Noncomplex chronic	7332 (26.7)	1270 (22.0)	1670 (28.9)	1994 (34.5)	1491 (25.8)
Complex chronic	6884 (25.1)	399 (6.9)	736 (12.7)	1500 (25.9)	3442 (59.5)

Abbreviations: AMC, academic medical center; PMCA, Pediatric Medical Complexity Algorithm.

^a^ All attributed children includes those with zero cost during the performance year. Cost quartiles 1-4 exclude children with zero cost.

^b^ Includes American Indian, Asian, and Hawaiian or Pacific Islander.

#### Cost

There were 4157 attributed children and young adults (15.2%) who had zero cost in 2018. Of those, 2232 individuals (53.7%) remained continually enrolled in Medicaid during the performance year, compared with approximately 90% of children with any costs. The TCOC during the performance year for all attributed children was a median (IQR) of $347 ($107-$1123) (Table 2) and mean (SD) of $2992 ($16 808). Nearly half (46%) of costs were accounted for by 272 attributed children and young adults (1.0%). Compared with children and young adults in the lowest-cost quartile (Q1), those in the highest-cost quartile (Q4) were more likely to be ages 18 to 20 years (372 individuals [6.4%] vs 802 individuals [13.9%]), White non-Hispanic (1188 individuals [20.5%] vs 1627 individuals [28.1%]), and to live farther from the AMC (median [IQR], 6 [4.6-20.3] vs 13.9 [4.6-30.9] miles). Children and young adults eligible for Medicaid via the blind or disabled category were disproportionately represented in Q4 (1642 individuals [28.4%]) compared with the other quartiles (eg, Q1: 181 individuals [3.1%]). The proportion of individuals with complex chronic medical conditions was highest in Q4 (3442 individuals [59.5%]) and lowest in Q1 (399 individuals [6.9%]).

**Figure 2.**
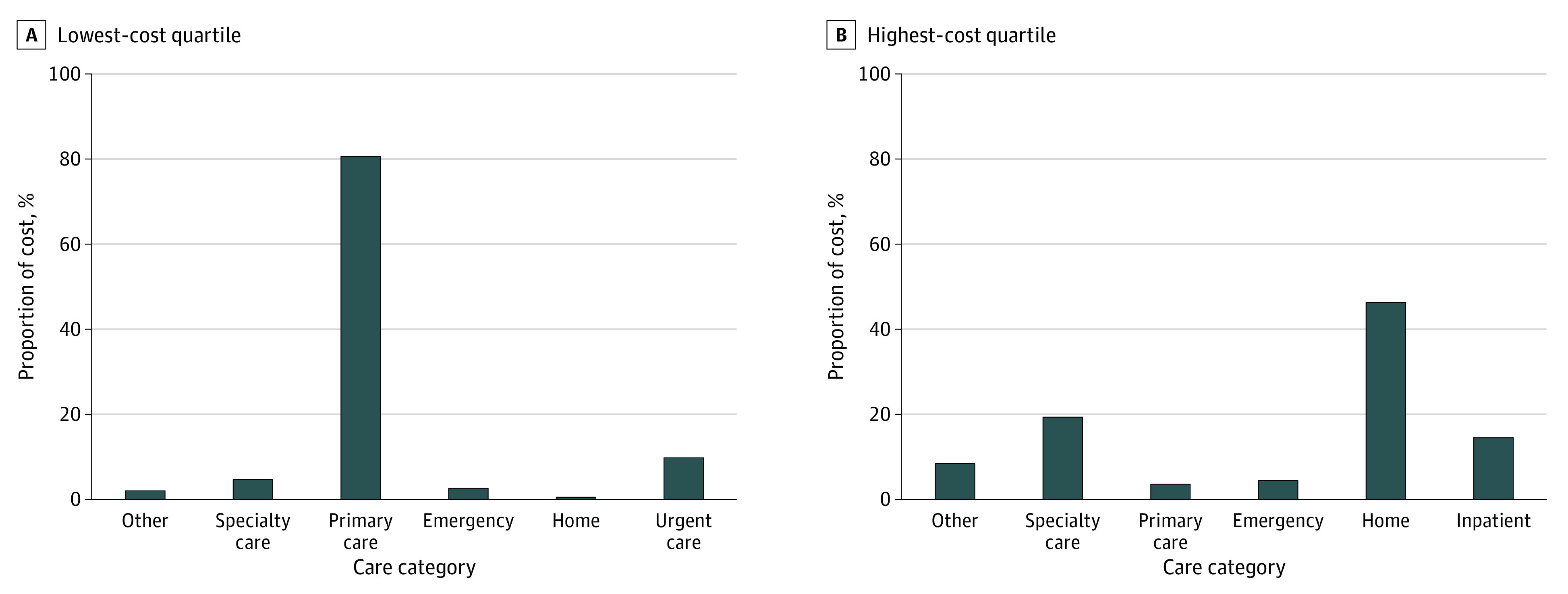
Health Care Utilization Patterns in the Accountable Care Organization Performance Year Among Attributed Children and Young Adults in Lowest and Highest Cost Quartiles

Clinical encounters in 4 settings accounted for most costs: home care (43% of TCOC), outpatient specialty care (19% of TCOC), inpatient care (14% of TCOC), and primary care (8% of TCOC). Most individuals received primary care during the performance year (87% in Q1, 92% Q2, 95% Q3, and 94% Q4). Children in Q1, Q2, and Q3 had no inpatient care. Among children in each cost quartile, the distribution of total annual expenditures among clinical settings is shown in Figure 2. For children in Q4, nearly half (47%) of TCOC was accounted for by home care services. Remaining expenditures included outpatient specialty care (20%), inpatient care (15%), emergency care (5%), and primary care (4%).

### Sensitivity Analyses

#### Attribution

We assessed varied attribution scenarios (eTable 1 in Supplement 1). Compared with prospective attribution, retrospective attribution resulted in a 50% larger attributed population (40 093 individuals vs 27 290 individuals) but lower mean total per member per month (PMPM) cost ($214 vs $279). We built on the prospective and retrospective attribution approaches with geography and cost-based criteria. Geographic assignment (individuals living in the AMC county with no claims in the attribution period were assigned to the ACO) led to substantially lower PMPM and far more enrollees in both prospective and retrospective approaches. Cost-based inclusion included individuals with claims of at least $100 000 and 75% of cost incurred within the ACO. This approach is relevant for AMCs that serve medically complex populations who may receive primary care at other locations. Cost-based inclusion approaches yielded similar results to prospective and retrospective attribution approaches alone.

#### Prescription Drugs

Prescription costs (mean, $1499.75; median, $24.70) ranged widely ($0-$2.1 million) (eTable 2 in Supplement 1). Children and young adults in Q4 had substantially higher prescription costs (mean [SD] $5262 [$52 448]; median [IQR], $285 [$40-$1520]) compared with those in lower cost quartiles (eg, Q3: mean [SD], $1134 [$12 735]; median [IQR], $110 [$17-$489]). Prescription costs contrasted between individuals in the top 1% of total cost (median [IQR], $5018.75 [$1436.66-$17 518.67]) and the top 10% of total cost (median [IQR], $482.20 [$71.01-$2847.10]).

#### Outlier Exclusion

We assessed the exclusion of children and young adults with the highest costs (eTable 3 in Supplement 1) as most ACOs include provisions to protect against catastrophic costs and reduce health care organization reluctance to participate in ACOs. The mean PMPM cost was reduced by 45% ($279 to $147) when excluding children and young adults with the top 1% of costs, and 79% ($279 to $55) when excluding children and young adults with the top 10% of costs (eTable 4 in Supplement 1).

### Health Care Outside the ACO

**Figure 3.**
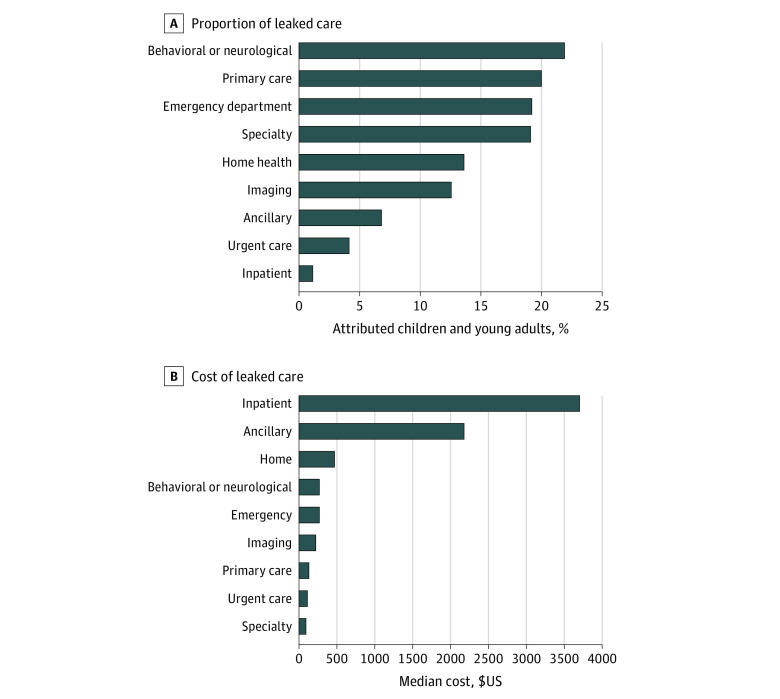
Type of Care Sought Outside the Accountable Care Organization (Leaked Care) During the Performance Year by Frequency and Cost

More than half (14 574 individuals [53.4%]) of attributed children and young adults had at least 1 health care encounter outside the ACO, including 2474 children (9% attributed population) whose care exclusively occurred outside the ACO. Leaked care varied by service: approximately 20% of the attributed population received care from a non-ACO clinician for behavioral health care (5972 individuals), primary care (5468 individuals), emergency care (5236 individuals), or specialty care (5211 individuals) (Figure 3A). Among attributed children and young adults receiving the following clinical services, most received leaked care: home health (99% of those who received home health care received leaked care), emergency department (75%), and behavioral health (72%). More than one-fourth (26%) of attributed children and young adults receiving primary care received at least some of their primary care outside the ACO.

Among 14 574 children with leaked care encounters, the total median (IQR) cost of non-ACO care was $348.97 ($130.40-$1326.21) per individual per year. Inpatient care and ancillary services were the highest-cost leaked care services (Figure 3B). The median (IQR) annual TCOC for children and young adults in Q4 for non-ACO care was $3845 ($2166-$7929), compared with $81 ($50-$103) for children in Q1 of non-ACO care. Children with complex chronic disease made up 65% of Q4 of non-ACO encounters, compared with 16% in Q1.

## Discussion

This cohort study used Medicaid claims data to simulate attribution to a hypothetical pediatric ACO and describe baseline cost and utilization patterns to inform future pediatric ACO design. As described nationally,^27,28,29^ 2 subpopulations of children and young adults were present in the attributed population. Most children were healthy, lived locally, and had low costs. A smaller population with higher medical complexity traveled farther for care and had higher expenditures. We found distinctive cost and utilization patterns both within and outside the ACO. Nearly all children and young adults received primary care, with few requiring inpatient hospitalization. Key drivers associated with high-cost care included home health care, outpatient specialty services, and inpatient care. Over half of attributed children and young adults received care outside of the ACO, particularly those receiving home health, emergency department, and behavioral health care. Our findings suggest considerations for pediatric ACO design regarding attribution, accounting for children and young adults with high costs, and strategies to address high levels of leaked care.

Attribution choices can drive care management strategies for pediatric ACOs. While prospective attribution allows clinicians to know which children they are accountable for ahead of time, in this analysis, nearly 15% of prospectively attributed children had no claims and nearly 15% were not continually enrolled in Medicaid during the performance year. Consistent primary care is a key component of pediatric ACO success, with longer-term attribution and engagement leading to improved outcomes and decreased cost.^17,30,31^ For pediatric ACOs using prospective attribution, outreach to engage individuals missing primary care and continuous enrollment strategies, such as automatic renewal mechanisms, presumptive enrollment, or enhanced benefits to stimulate engagement (eg, value-added services), should be considered.^32,33^ Alternative attribution methods resulted in different populations and utilization profiles. Each approach will have unique ACO performance tradeoffs, depending on local context and ACO goals. For example, retrospective methods may mitigate financial impacts of churn or lack of continuity, but targeting interventions might be challenging, given uncertainty around attribution. Decision-makers should carefully consider the importance of consistent primary care and known disparities in primary care engagement among children in communities that have been historically marginalized to achieve success in pediatric ACOs.

Only 1% of attributed children and young adults accounted for nearly half of costs. This group had higher medical complexity and was more geographically dispersed than children and young adults with lower costs, likely traveling to the AMC for care, given geographic distribution gaps in subspecialty care.^34^ National data suggest that this population is comprised of children with rare, complex conditions, such as congenital or neurologic diseases, requiring care from multiple subspecialists.^28^ The care utilization phenotypes of this group have implications for ACO care design. Compared with adults, children with the highest expenditures use more primary care, home-based care, and specialty outpatient care, and require fewer hospitalizations.^9^ Instead of focusing on postacute care utilization, like adult-focused ACOs, pediatric ACOs might consider promoting coordination and data-sharing among subspecialists at AMCs and geographically dispersed primary care physicians and improving access and quality of pediatric home-based care.

The clinical profiles of children with the highest expenditures also have implications for risk adjustment and high-cost outlier situations. Risk-adjustment methodologies are used to account for clinical severity when determining financial benchmarks for ACOs^35^ and are important for minimizing payer screening of higher-cost patients. Most risk-adjustment models are optimized based on mixed populations of adults and children, up-weighting proportionally larger diagnoses and utilization profiles of adults (eg, heart disease, diabetes).^36^ Recalibration of models to include factors important for children, such as geography or PMCA score, as well as consideration of unique pediatric conditions or settings (eg, home care, pediatric intensive care) could impact pediatric ACO benchmarks. Additionally, exclusion thresholds for high-cost outliers, such as unpredictable catastrophic expenses, can impact ACO success.^37,38^ While it has been suggested that children with complex conditions^9,20^ be excluded from ACO responsibility, factors such as home-based care or prescription drug costs could add nuance when determining exclusion thresholds. While MSSP ACOs exclude the top 1% of patients nationally by eligibility category, there are no guidelines for outlier exclusion in pediatrics.^31^ Here, exclusion of children and young adults in the top 1% of cost reduced cost by nearly 50%, suggesting that this criterion may be sufficient to protect pediatric ACOs against unpredictable cost.

More than half of attributed children and young adults sought care outside of the ACO, at a median cost of $349 per child, which is substantial when the median TCOC was $347. Leaked primary care, while less frequent in adult ACOs compared with our findings (8% vs 20%), is associated with higher spending and lower quality of care among ACOs disproportionately caring for beneficiaries from racial or ethnic minority groups, such as Black and Hispanic or Latinx individuals.^39^ Leakage can also weaken the hypothesized benefits of systematic coordinated care with incentive alignment. ACOs may elect to avoid taking on full risk if there are strong possibilities of leakage that can lead to uncontrollable costs and outcomes. Adult ACOs have paid particular attention to managing costly leakages in specialty care, using strategies such as modifying attribution approaches to optimize beneficiary assignment, creating electronic consultation systems to limit referrals, increasing copays for non-ACO encounters, or even limiting clinician consolidation into multispecialty organizations.^40,41^ In this study, the high frequency and cost of pediatric leaked care highlights the need for analogous pediatric-specific strategies to enhance within-ACO care coordination.^39^

The costliest leaked encounters comprised inpatient, home health, and ancillary service encounters, reflecting the disproportionate representation of children with medical complexity. However, the most frequently leaked care included behavioral health, primary care, and emergency care. Children with neurodevelopmental conditions, such as autism spectrum disorder, often require frequent home- and community-based mental health care.^41^ Traditionally, agencies and individuals providing these services are not employed in health systems, but pediatric ACOs can build alignments with these agencies. Such partnerships can facilitate cross-sector communication, timely care delivery, and potentially lead to shared financial accountability. The large amount of primary care leakage observed in this study also suggests a need for community-based strategies that account for social needs, such as lack of transportation. Pediatric ACOs can employ care coordinators or community health workers to help families navigate fragmented services.

As seen from the few children with high costs and the overall low TCOC in this study, opportunities for cost containment are comparatively few for pediatric ACOs compared with adult ACOs. Successful implementation of pediatric ACOs that promote health for the children without medical complexity will require an emphasis on prevention and development, in alignment with general pediatric care. Given the clear impacts of early-life poverty, structural racism, and adverse childhood experiences on toxic stress and health disparities, Medicaid ACOs should consider interventions that promote health and resilience in the context of adversity (eg, postpartum depression screening, Healthy Steps, social needs screening).^42^ As short-term cost savings will likely be smaller than in adult ACOs, pediatric quality measures can place increased weight on vetted measures, such as well-child checks and social-emotional screening, while piloting financial structures to account for the long-term benefits of these interventions.^43^ To support continued evolution of pediatric ACOs, further research assessing the longitudinal and cross-sector (eg, education) benefits of preventive care and integration of medical and social care is needed.

### Limitations

Our study has some limitations. We used claims data with an aim to generate actionable insight for future pediatric ACOs. The findings provide a baseline to inform design strategies unique to pediatric populations, but local context (eg, state payment reforms, participation in ACOs) will influence application. This was a cross-sectional analysis at a single AMC in 1 state’s Medicaid program, so results may not be generalizable. Similar to other AMCs, the AMC here comprises a teaching hospital with a children’s hospital and a network of statewide outpatient facilities. While AMCs account for only 5% of hospitals nationwide, they provide a disproportionate share of care for Medicaid-enrolled children.^44^ Given the nascent state of pediatric ACOs, our findings add to the limited evidence to inform pediatric ACO design for Medicaid-enrolled children and young adults and may be particularly relevant for AMCs. The AMC here does not include safety-net organizations, such as federally qualified health centers, which can account for up to one-fourth of pediatric Medicaid primary care visits. Unique barriers to safety-net organizations’ ACO participation, such as siloed funding streams and regulatory requirements, need further exploration.^45^ We used 1 year of claims data for attribution and 1 year to analyze performance, similar to approaches used by many Medicaid ACOs. While findings could be outliers, given the relatively short time period, it is reassuring that the patterns observed align with data from pediatric Medicaid populations in other states.^9,46^ We chose our attribution method to most closely simulate planned attribution under value-based payment arrangements in the state Medicaid program, which heavily weights primary care. Many existing ACOs, including MSSP, use plurality of primary care in attribution algorithms so that the patient is attributed to the physician who provided most of the patient’s primary care.^26^ We did not have sufficient information to assign NPIs outside of the AMC, so it is possible that some individuals who had less than 50% of their primary care spending at the AMC could still have had the greatest proportion (plurality) of their primary care at the AMC. While we could identify those with most (>50%) of their care at the AMC, we were not able to determine where the plurality of care took place.

## Conclusion

In this cohort study, we offer practical evidence by delineating cost and utilization patterns in a pediatric population attributed to a hypothetical ACO under a number of different potential scenarios and highlight opportunities that can be leveraged to improve child health through future pediatric ACO design. There is increasing momentum around pediatric-focused ACOs and APMs, but to fully realize potential benefits for children, pediatric ACO design cannot simply mimic adult APM strategies.

## Notes

Supplement 1. **eTable 1.** Specifications of Attribution Methods Considered in Sensitivity Analyses **eTable 2.** Comparisons of Attribution Specifications **eTable 3.** Sensitivity Analysis: Prescription Drug Costs by Cost Quartile **eTable 4.** Outlier Exclusion of Top 1% or Top 10% Costs

Supplement 2.
Data Sharing Statement
