# Supplementary material for: Analysis of North Carolina Medicaid Claims Data to Simulate a Pediatric Accountable Care Organization
Source: JAMA Netw Open. 2023 Aug 4;6(8):e2327264. doi: 10.1001/jamanetworkopen.2023.27264 (PMC10403786; doi:10.1001/jamanetworkopen.2023.27264)
Supplement: Supplement 1. — eTable 1. Specifications of Attribution Methods Considered in Sensitivity Analyses eTable 2. Comparisons of Attribution Specifications eTable 3. Sensitivity Analysis: Prescription Drug Costs by Cost Quartile eTable 4. Outlier Exclusion of Top 1% or Top 10% Costs [file jamanetwopen-e2327264-s001.pdf]

Supplemental Online Content

Cholera R, Anderson DM, Chung R, et al. Analysis of North Carolina Medicaid claims data to simulate a pediatric accountable care organization. *JAMA Netw Open*. 2023;6(8):e2327264. doi:10.1001/jamanetworkopen.2023.27264

eTable 1. Specifications of Attribution Methods Considered in Sensitivity Analyses

eTable 2. Comparisons of Attribution Specifications

eTable 3. Sensitivity Analysis: Prescription Drug Costs by Cost Quartile

eTable 4. Outlier Exclusion of Top 1% or Top 10% Costs

This supplemental material has been provided by the authors to give readers additional information about their work.

© 2023 Cholera R et al. *JAMA Network Open.*

eTable 1. Specifications of Attribution Methods Considered in Sensitivity Analyses

	Alternative A (Prospective)	Alternative B (Retrospective)	Alternative C (Prospective/High-Cost)	Alternative D (Retrospective/High-Cost)	Alternative E (Prospective/ Geographic)	Alternative F (Retrospective/ Geographic)
Prospective or retrospective attribution for primary care?	Prospective	Retrospective	Prospective	Retrospective	Prospective	Retrospective
Include children with significant cost at AMC?	*[No]*	*[No]*	>$100,000 allowed claims (75% at ACO)	>$100,000 allowed claims (75% at ACO)	*[No]*	*[No]*
Include children based on geography?	*[No]*	*[No]*	*[No]*	*[No]*	Resident of ACO home county	Resident of ACO home county

Prospective attribution (Alternative A) occurs prior to the performance year and was the attribution method used in the primary anlysis. **Retrospective attribution (Alternative B)** occurs concurrently with the performance year. The other methods build on these first two methods. **Cost-based attribution (Alternatives C and D)** additionally attributes individuals who had at least $100,000 in allowed Medicaid claims and had at least 75% incurred at the ACO; this method was included as AMCs may serve patients with high costs and complex medical needs, but who receive primary care services at other locations. **Geographic inclusion (Alternatives E and F)** adds geography-based inclusion by assigning individuals to the ACO who lived in the county where the AMC is based, but who had no claims in the attribution period.

© 2023 Cholera R et al. *JAMA Network Open.*

eTable 2. Comparisons of Attribution Specifications

	Alternative A	Alternative B	Alternative C	Alternative D	Alternative E	Alternative F
Number of patients	27,290	40,918	27,325	40,944	40,083	54,517
**Primary Care Costs in 2018**						
Total cost, Primary Care claims						
Mean (SD)	249.18 (592.41)	321.40 (555.54)	250.21 (594.23)	321.71 (556.37)	195.09 (508.56)	244.33 (499.81)
95% CI	242.15 - 256.21	316.02 - 326.79	275.94 - 286.60	316.32 - 327.11	190.11 - 200.07	240.14 - 248.53
Total Cost of any care, PMPM						
Mean (SD)	278.79 (2,521.10)	240.89 (1,459.54)	305.58 (2,794.28)	262.26 (2,362.82)	248.11 (2,491.52)	214.81 (1,626.80)
95% CI	248.01 - 308.95	226.75 - 255.04	271.54 - 339.04	239.36 - 285.15	222.68 - 273.13	201.15 - 228.47
Zero total cost	4,157 (15.2%)	0 (0.0%)	4,157 (15.2%)	0 (0.0%)	10,164 (25.4%)	8,026 (14.7%)
95% CI	14.8% - 15.7%		14.8% - 15.6%		24.9% - 25.8%	14.4% - 15.0%
**Demographics**						
Female	13,694 (50.2%)	20,532 (50.2%)	13,717 (50.2%)	20,547 (50.2%)	20,015 (49.9%)	27,211 (49.9%)
	49.6% - 50.8%	49.7% - 50.7%	49.6% - 50.8%	49.7% - 50.7%	49.4% - 50.4%	49.5% - 50.3%
Age, years						
Mean (SD)	10.3 (5.4)	10.2 (5.2)	10.3 (5.4)	10.2 (5.2)	11.0 (5.6)	10.8 (5.5)
95% CI	10.2 - 10.4	10.1 - 10.2	10.2 - 10.4	10.1 - 10.2	10.9 - 11.1	10.8 - 10.9
Race						
White	12,623 (46.3%)	19,951 (48.8%)	12,642 (46.3%)	19,967 (48.8%)	17,175 (42.8%)	24,905 (45.7%)
	45.7% - 46.9%	48.3% - 49.3%	45.7% - 46.9%	48.3% - 49.3%	42.4% - 43.3%	45.3% - 46.1%
Black	12,748 (46.7%)	17,687 (43.2%)	12,762 (46.7%)	17,696 (43.2%)	20,045 (50.0%)	25,411 (46.6%)
	46.1% - 47.3%	42.8% - 43.7%	46.1% - 47.3%	42.8% - 43.7%	49.5% - 50.5%	46.2% - 47.0%
Ethnicity						
Hispanic/Latinx	6,308 (23.1%)	11,085 (27.1%)	6,315 (23.1%)	11,091 (27.1%)	9,935 (24.8%)	15,017 (27.5%)
	22.6% - 23.6%	26.7% - 27.5%	22.6% - 23.6%	26.7% - 27.5%	24.4% - 25.2%	27.2% - 27.9%
Urban/Rural	23,885 (87.6%)	37,154 (90.8%)	23,910 (87.5%)	37,174 (90.8%)	36,678 (91.5%)	50,753 (93.1%)
Urban	87.2% - 88.0%	90.6% - 91.2%	87.2% - 88.0%	90.6% - 91.1%	91.3% - 91.8%	92.9% - 93.4%
Distance from AMC, miles						
Mean (SD)	19.6 (59.6)	23.7 (59.2)	19.7 (59.6)	23.7 (59.2)	19.5 (83.1)	22.3 (77.9)
95% CI	18.9 - 20.3	23.0 - 24.1	19.0 - 20.4	23.0 - 24.1	18.7 - 20.4	21.4 - 22.7

© 2023 Cholera R et al. *JAMA Network Open.*

	Alternative A	Alternative B	Alternative C	Alternative D	Alternative E	Alternative F
Continually Enrolled (2018)	23,582 (89.7%)	36,658 (89.6%)	23,616 (89.7%)	36,681 (89.6%)	32,737 (87.3%)	46,505 (85.3%)
	89.3% - 90.0%	89.3% - 89.9%	89.3% - 90.1%	89.3% - 89.9%	87.0% - 87.7%	85.0% - 85.6%

© 2023 Cholera R et al. *JAMA Network Open.*

eTable 3. Sensitivity Analysis: Prescription Drug Costs by Cost Quartile

	Overall	0 cost	Quartile 1 cost	Quartile 2 cost	Quartile 3 cost	Quartile 4 cost
Number of patients	27,290	4,157	5,782	5,784	5,784	5,783
**Prescription Costs ($)**						
Median (IQR)	24.70 (0 - 294.53)	0.00 (0 - 0)	0.00 (0.00 - 41.37)	33.84 (0 - 211.10)	110.29 (17.38 - 488.61)	285.18 (40.03 - 1,519.53)
Mean (SD)	1,499.75 (25,138.78)	10.90 (256.76)	162.63 (646.25)	510.35 (7,106.07)	1,134.27 (12,734.56)	5,262.01 (52,448.09)
Range	0.00 - 2,142,658.21	0.00 - 10,893.26	0.00 - 16,066.80	0.00 - 405,265.87	0.00 - 523,139.47	0.00 - 2,142,658.21

© 2023 Cholera R et al. *JAMA Network Open.*

eTable 4. Outlier Exclusion of Top 1% or Top 10% Costs

	All Attributed Children	Exclude Top 1% of Children with Highest Cost	Exclude Top 10% of Children with Highest Cost
Number of patients	27,290	27,018	24,561
Total cost, PBPY ($)			
Mean (SD)	2,992.47 (16,808.26)	1,628.52 (4,517.68)	602.25 (814.58)
Median (IQR)	347.24	340.74	283.01
Total PMPM ($)			
Mean (SD)	278.79 (2,521.10)	146.64 (406.99)	55.42 (91.29)
Range	0.00 - 325,676.62	0.00 - 8,774.22	0.0 - 6,912.89

Excluding top 1% would only exclude 272 children, and decrease mean costs by $1,363.95 PBPY (~46% less). Excluding top 10% would decrease mean costs by $2,390.22 (~80% less), but excludes 2,729 children.

© 2023 Cholera R et al. *JAMA Network Open.*
