# Supplementary material for: Analysis of North Carolina Medicaid Claims Data to Simulate a Pediatric Accountable Care Organization
Source: JAMA Netw Open. 2023 Aug 4;6(8):e2327264. doi: 10.1001/jamanetworkopen.2023.27264 (PMC10403786; doi:10.1001/jamanetworkopen.2023.27264)
Supplement: Supplement 2. — Data Sharing Statement [file jamanetwopen-e2327264-s002.pdf]

Data Sharing Statement

Cholera. Analysis of North Carolina Medicaid Claims Data to Simulate a Pediatric Accountable Care Organization. *JAMA Netw Open*. Published August 04, 2023. doi:10.1001/jamanetworkopen.2023.27264

Data
Data available: No

Additional Information
Explanation for why data not available: This data is governed by a data use agreement between NCDHHS and Duke University.
